# Acute Medial Collateral Ligament With Postero-Medial Complex Repair in Multiligament Knee Injuries Using a Novel “Double Shoelace Repair” Technique: 10-Year Clinical and Functional Outcomes

**DOI:** 10.7759/cureus.102328

**Published:** 2026-01-26

**Authors:** Jagadeesh P Chamundaiah, Nirav R Gupta, Syed A Mubdi, John Edwin, Senthilkumar Mahalingam

**Affiliations:** 1 Department of Orthopaedics, Kauvery Hospital, Electronic City, Bengaluru, IND; 2 Department of Orthopaedics, Apex Superspeciality Hospital, Mumbai, IND; 3 Department of Orthopaedics, Basildon University Hospital, Basildon, GBR; 4 Department of Orthopaedics, Royal Cornwall Hospital, Truro, GBR

**Keywords:** acute mcl repair, double shoelace repair, ikdc score, lysholm score, medial collateral ligament injury, mlki, multiligamentous knee injuries, postero-medial complex injury

## Abstract

Background: Femoral-sided medial collateral ligament (MCL) avulsion and posteromedial corner (PMC) injury frequently occur together in the setting of multiligament knee injuries (MLKIs). Untreated PMC injuries are prone to develop residual valgus and rotatory instability. Early anatomic repair optimises outcomes. This study evaluates the long-term results of a novel “double shoelace repair” technique for acute MCL and PMC injuries performed with concurrent cruciate reconstructions.

Methods: A retrospective analysis was conducted on patients with MLKI who underwent acute (less than three weeks old) femoral-sided MCL and PMC repair using the double shoelace construct between 2011 and 2014. Concomitant anterior cruciate ligament (ACL)/posterior cruciate ligament (PCL) injuries were treated with single-stage all-inside reconstructions. Demographic data, injury patterns, stability, complications, and functional outcomes (Lysholm Knee Scoring Scale, International Knee Documentation Committee assessment, Tegner Activity Scale) were recorded. Minimum follow-up was 10 years. Statistical significance was set at p < 0.05.

Results: Forty-one patients (mean age 35.12 ± 11.64 years) were included. The most common injury pattern was ACL + MCL + PMC (78.05%). Mean time to surgery was 6.95 days, and mean follow-up was 12.43 years. Lysholm scores improved from 27.27 ± 9.4 to 92.8 ± 2.84 (p < 0.001) and IKDC scores from 37.2 ± 6.8 to 88.78 ± 4.91 (p < 0.001). The mean Tegner score at final follow-up was 4.54 ± 1.52. Of 10 competitive athletes, nine (90%) returned to preinjury level at a mean of 10.4 months. Complications were minimal: two failures (4.88%), no arthrofibrosis, and transient sensory deficits in 12.19%.

Conclusion: The double shoelace technique provides dependable restoration of medial stability and excellent long-term function in acute MCL + PMC injuries associated with MLKIs, with high return-to-sport rates and low complication rates. Further comparative studies are needed to refine indications.

## Introduction

The medial collateral ligament (MCL) is among the most frequently injured ligaments of the knee, with an estimated incidence ranging from 0.24 to 7.3 per 1000 individuals [1]. Of its two anatomical components, the superficial MCL (sMCL) is injured more commonly and serves as the primary restraint to valgus stress throughout knee flexion [2]. The posterior oblique ligament (POL), a key stabiliser within the posteromedial corner (PMC), is anatomically and functionally distinct from the sMCL, as it provides primary restraint to internal tibial rotation and contributes secondarily to valgus stability up to 30° of knee flexion [2,3].

Injuries to the PMC frequently result from rotational mechanisms and are commonly associated with concomitant anterior cruciate ligament (ACL) or posterior cruciate ligament (PCL) injuries. Multiligament knee injuries (MLKIs) are classified using Schenck Classification [4], which is as follows: (i) Knee dislocation (KD) I, the least severe form, with tear of either cruciate ligament (ACL or PCL), (ii) KD II dislocations are tears of both the cruciate ligaments (ACL and PCL), KD III involves tear of both cruciates and any one collateral ligament, with subclassification of KD IIIM which includes tears of the ACL, PCL, and MCL, while KD IIIL includes tears of the ACL, PCL, and lateral collateral ligament (LCL), and (iv) KD IV is characterized by tears of all the four ligaments and is generally associated with neurovascular injuries.

While isolated MCL injuries are generally managed conservatively, considerable debate persists regarding the optimal management strategy for MCL lesions occurring in the context of MLKI. Ongoing controversy surrounds indications for operative versus non-operative treatment, timing of surgery (acute versus delayed), and the preferred surgical approach (repair versus reconstruction). Current evidence suggests that acute repair is advantageous for ligament avulsion injuries at the femoral or tibial insertions due to their reduced intrinsic healing potential [5-7].

In this study, we describe a novel surgical approach, the “double shoelace repair” technique, for acute femoral-side MCL avulsion with associated PMC injury in the setting of MLKI. We aim to evaluate whether early repair using this method can achieve functional outcomes comparable to existing treatment strategies for MCL repair in patients with MLKI.

## Materials and methods

This retrospective study was carried out at Kauvery Hospital, Electronic City, Bengaluru, India, and included all eligible patients operated on by a single senior arthroscopy surgeon between January 2011 and December 2014.

Study population

**Figure 1:**
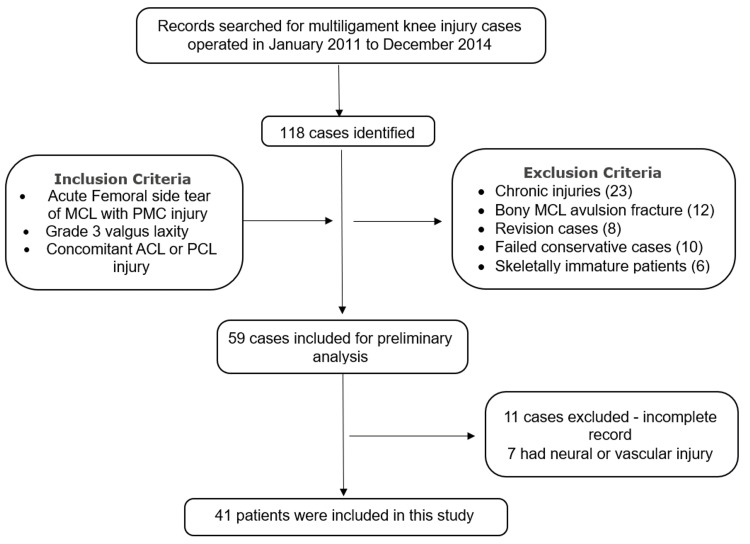
Flow diagram of study population for double shoelace repair of acute femoral-sided MCL with PMC injuries MCL: medial collateral ligament; PMC: posteromedial complex; ACL: anterior cruciate ligament; PCL: posterior cruciate ligament

Inclusion criteria were patients with acute (less than three weeks old) femoral-side MCL tears associated with Grade three valgus laxity, anteromedial rotatory instability (AMRI), and concomitant ACL or PCL injuries. The three-week interval was considered a critical period during which soft-tissue planes remain adequately defined, allowing anatomical repair without significant scarring. Exclusion criteria included the presence of neurovascular injury, chronic cases (more than three weeks old), prior failed or revision surgeries around the knee, and unfused physis (Figure 1). 

Data collection

Preoperative, intraoperative, and follow-up information were obtained retrospectively from patient medical records. Data regarding the pattern of ligament involvement, time since injury, preoperative limb alignment, postoperative valgus stability, and functional outcome scores such as the Lysholm Knee Score (LKS) [8], International Knee Documentation Committee (IKDC) score [8], and Tegner score [8] were extracted.

The LKS is a simple questionnaire-based scoring system used to evaluate the outcomes following knee ligament surgeries, particularly symptoms of instability. It includes the following eight items: limp, use of support while walking, locking sensation, giving way sensation (feel of instability), pain, swelling, stair climbing, and squatting. The final score is categorised as excellent (95-100), good (84-94), fair (65-83), and poor (≤64) [8].

The IKDC score is a subjective scoring system used to detect improvement or deterioration due to knee impairment. It has three main domains: (i) Symptoms (seven items), (ii) Sports (one item) and daily activity (nine items), and (iii) current knee function (one item). The possible score range is 0-100, with 100 indicating no limitations in sports and daily activities and no symptoms [8].

The Tegner score was developed as a complement to LKS. It includes a graduated list of 11 activities of daily living, recreation, and competitive sports. The patients pick one out of the list of 11 that best describes their current level of activity. A score of 0 indicates sick leave or disability due to the knee, and a score of 10 indicates national or international competitive sports participation. A score of 6-10 can only be achieved if the patient participates in some form of sports activity [8]. All patients had a minimum clinical and radiological follow-up of 10 years.

Surgical procedure

Each patient was positioned supine under spinal anaesthesia with the knee flexed to 90°, supported by a lateral post and footrest. After standard preparation and draping, diagnostic arthroscopy was performed, and associated intra-articular pathologies, including ligamentous or meniscal injuries, were treated. ACL and/or PCL tears were addressed first with all-inside reconstruction using hamstring autografts primarily, and in cases where both cruciate ligaments required reconstruction, a peroneus longus graft was additionally harvested. Femoral-side graft fixation was performed initially, while tibial fixation was deferred until after the MCL and PMC repair.

**Figure 2:**
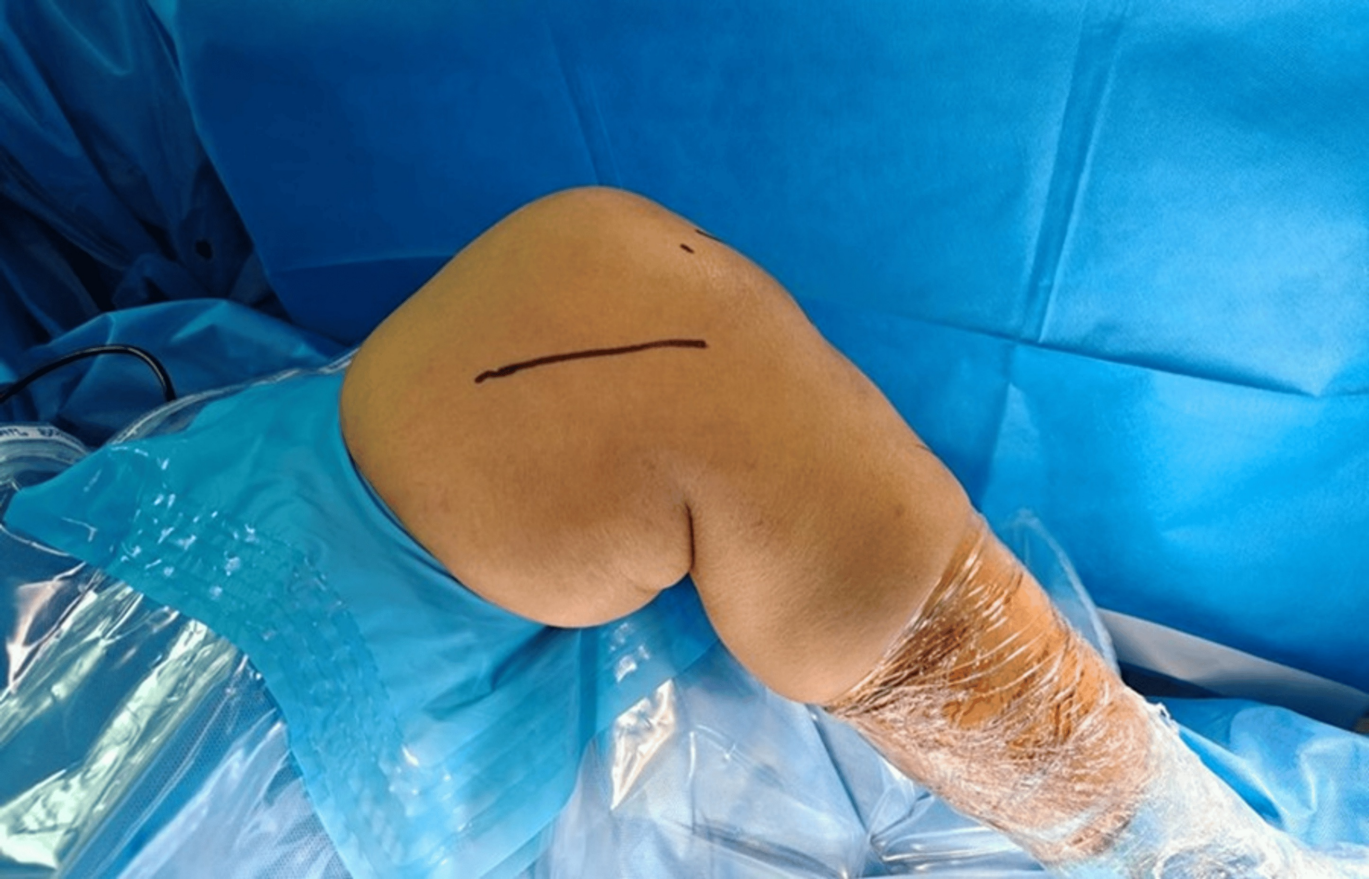
Intra-operative position of the knee with surface marking of the incision site on the medial side. Image Credit: Author

For the open medial approach, anatomical landmarks including the adductor tubercle, medial epicondyle, medial patellar facet, and joint line were identified. With the knee flexed at 90°, a 5-7 cm longitudinal incision was made beginning approximately 2 cm proximal to the medial epicondyle and extending to the medial joint line, 4-5 cm medial to the medial patellar facet (Figure 2). 

**Figure 3:**
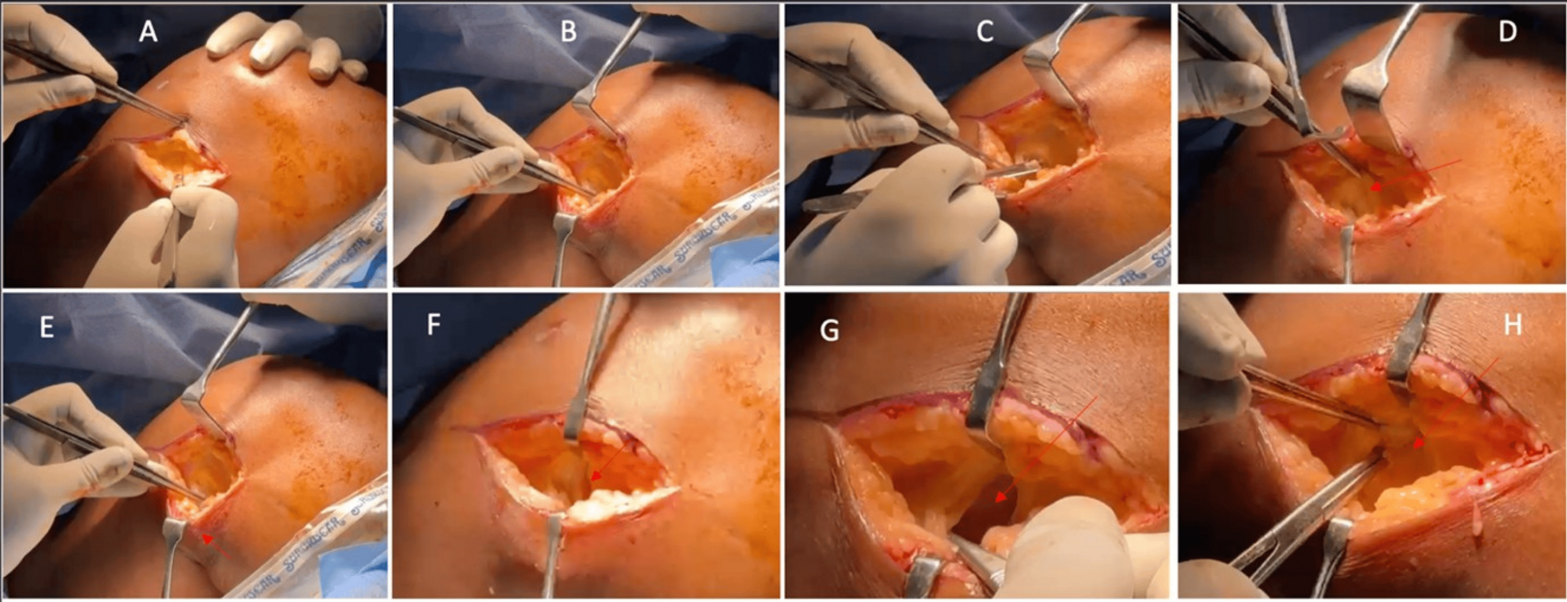
Intraoperative images demonstrating the sequential dissection. (A, B) Superficial and deep dissection; (C, D) Incision of the sartorial fascia, followed by posterior retraction of the hamstring tendons with sartorius (E) and anterior retraction of the medial parapatellar tendon (F); (G) Joint cavity; (H) Torn MCL (arrow) held with forceps. Image Credit: Author

Dissection proceeded carefully through the subcutaneous tissue to the sartorial fascia, with meticulous attention to protecting the infrapatellar branch of the saphenous nerve as it crosses anteriorly approximately 1 cm above the joint line. Following the creation of skin flaps, the sartorial fascia was incised in line with the skin incision, and the hamstring tendons were retracted posteriorly to expose the sMCL and POL. The medial femoral epicondyle was palpated, and the anatomical femoral insertion of the MCL was determined, approximately 3.2 mm proximal and 4.8 mm posterior to the epicondyle (Figure 3) [2]. 

**Figure 4:**
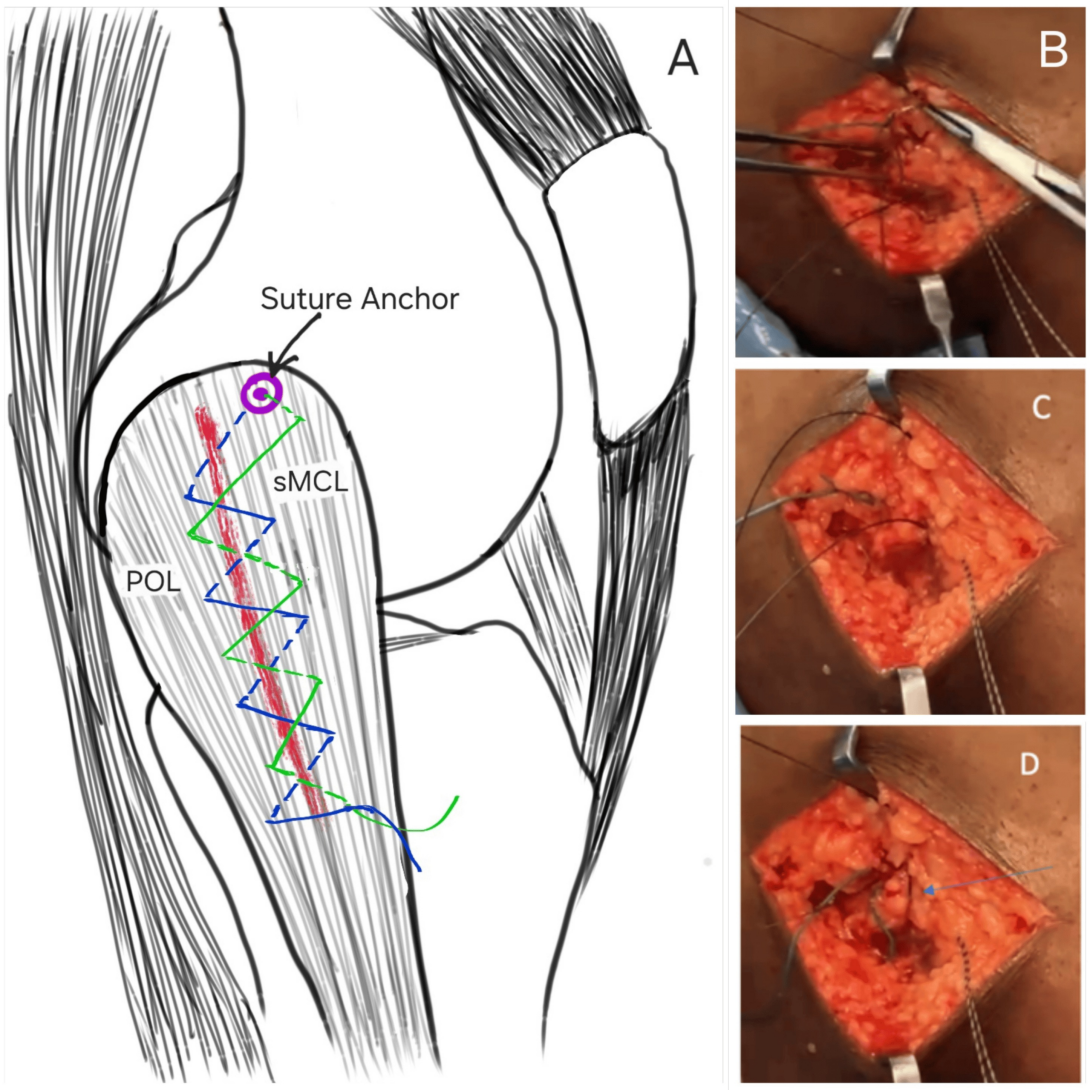
(A) Double shoelace repair technique; (B-D) Sequence of double shoelace repair sMCL: superficial medial collateral ligament; POL: posterior oblique ligament Image Credit: Author

This isometric point was decorticated to promote ligament healing, and a 5 mm double-loaded polyether ether ketone (PEEK) suture anchor was placed. Repair of the sMCL and POL was performed using a shoelace suture configuration from proximal to distal, advancing the anterior margin of the PMC to the sMCL and restoring tension across the posteromedial structures (Figure 4). 

**Figure 5:**
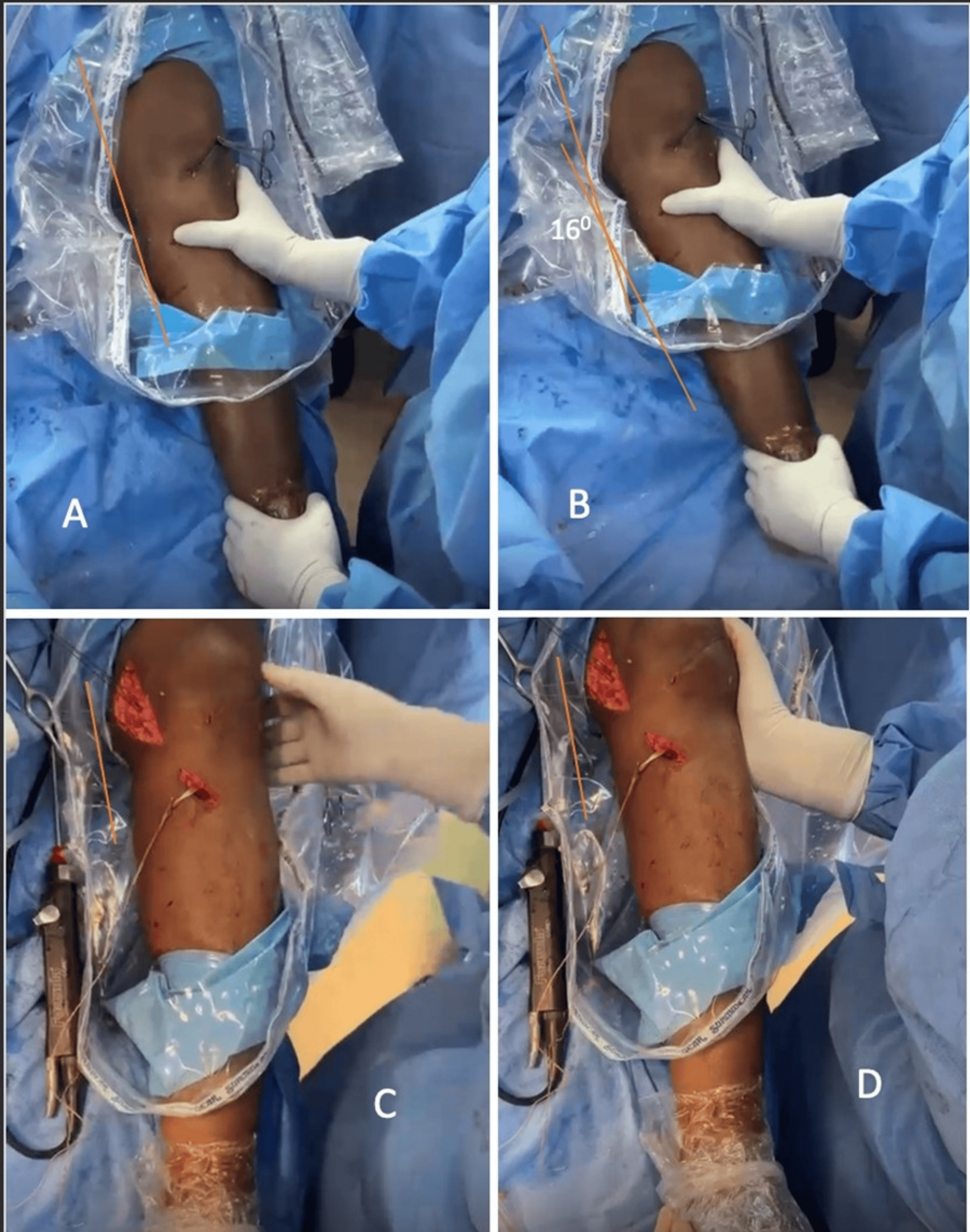
(A, B) Pre-operative valgus stress views, showing increased medial compartment opening; (C, D) Post-repair valgus stress views, with complete restoration of medial stability and no residual opening. Image Credit: Author

The same sutures were then passed distally to proximally in a second shoelace pattern to reinforce the repair, and knots were secured at 30° of knee flexion with neutral rotation and slight varus positioning. Stability was confirmed via intraoperative valgus stress testing (Figure 5). 

ACL/PCL tibial fixation was completed once the medial repair was stabilised. This “double shoelace” repair technique provides strong tissue anchorage and minimises the risk of suture cut-through due to broad contact distribution. Haemostasis was achieved, and wound closure was performed in layers without the use of drains.

Postoperatively, patients were placed in a hinged knee brace, and closed-chain flexion from 0° to 30° was initiated from the second postoperative day to prevent arthrofibrosis. Flexion beyond 60° was permitted after three weeks and flexion past 90° at six weeks. Patients remained non-weight-bearing for six weeks, and the brace was discontinued at twelve weeks. Return to sports activities was allowed once adequate quadriceps strength and neuromuscular control were restored.

Statistical analysis

Statistical analysis was conducted using JASP version 0.18.2.0 (University of Amsterdam, Netherlands) and Microsoft Excel 2013 (Microsoft Corporation, Redmond, Washington, United States). Descriptive statistics, including mean, standard deviation (SD), range, and percentage, were calculated for all variables. LKS and IKDC scores were reported as mean ± SD at preoperative assessment and at one-year, five-year, and 10-year follow-ups, while Tegner scores were documented at 10 years. Functional outcome changes were analysed using the Student’s t-test with significance set at p < 0.05.

## Results

A total of 41 patients met the inclusion criteria for this study, comprising 38 male (92.68%) and three female (7.32%) patients, with a mean age of 35.12 ± 11.64 years (range: 18-59 years). The most common mechanism of injury was road traffic accidents, followed by sports-related trauma (Table 1). 

**Table 1:** Demographics and injury characteristics of the study cohort

Demographic Parameter		Value
Age (years) mean ± SD (range)		35.12 ± 11.64 (18-59)
Gender n (%)	Male	38 (92.68)
	Female	3 (7.32)
Side n (%)	Right	24 (58.54)
	Left	17 (41.46)
Mode of Injury n (%)	Road Traffic Accident	27 (65.85)
	Sports injury	10 (24.39)
	Fall from Height	4 (9.76)

The majority of patients sustained combined ACL, MCL, and PMC injuries, accounting for 32 cases (78.05%). Four patients (9.76%) presented with PCL together with MCL and PMC injuries, while five patients (12.19%) demonstrated multiligament involvement, including ACL, PCL, MCL, and PMC. Based on the Schenck classification system, 36 knees (87.81%) were categorised as KD I and five knees (12.19%) as KD III-M. The mean interval from injury to surgical intervention was 6.95 ± 3.6 days (range 3-17), while the mean duration of follow-up was 12.43 ± 1.02 years (range 10.35-13.92). Two patients within the ACL + MCL and PMC group had associated meniscal injury, and one patient within the ACL + PCL + MCL and PMC group had an associated patellar dislocation (Table 2). 

**Table 2:** Injury pattern, classification and follow-up characteristics of the study cohort KD: knee dislocation; 3M: 3 medial; ACL: anterior cruciate ligament; PCL: posterior cruciate ligament; MCL: medial collateral ligament; PMC: posteromedial corner ^$^ two patients had meniscus injury, ^#^ one patient had patella dislocation

Injury Profile		Value
Ligaments Injured, n (%)	ACL + MCL and PMC	32^$^ (78.05)
	PCL + MCL and PMC	4 (9.76)
	ACL + PCL + MCL and PMC	5^#^ (12.19)
Schenck Classification, n (%)	KD 1	36 (87.81)
	KD 3 M	5 (12.19)
Duration from injury to surgery (days), mean ± SD (range)		6.95 ± 3.6 (3-17)
Follow-up (years), mean ± SD (range)		12.43 ± 1.02 (10.35-13.92)

**Figure 6:**
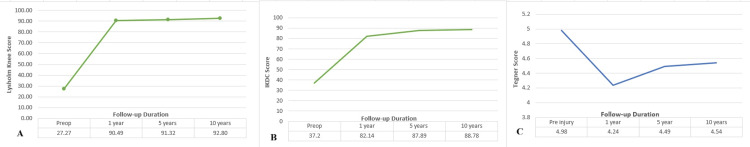
Trends in (A) Lysholm Knee Score, (B) IKDC scores, and (C) Tegner Score over 10 years of follow-up IKDC: International Knee Documentation Committee; preop: preoperative

Functional outcomes were evaluated using the LKS, IKDC assessment, and Tegner activity scale. The mean Lysholm score demonstrated significant improvement from 27.27 ± 9.4 preoperatively to 92.8 ± 2.84 at the final follow-up. Similarly, the mean IKDC score improved from 37.2 ± 6.8 preoperatively to 88.78 ± 4.91 at the final follow-up (Figure 6). 

The mean Tegner score at final follow-up was 4.54 ± 1.52. Among the 10 patients who participated in competitive sports preoperatively, nine (90%) successfully returned to their preinjury level, with a mean return-to-sport time of 10.4 ± 0.97 months.

Surgical failure was defined as valgus laxity greater than grade two at 30° of knee flexion or a positive anterior drawer test with external tibial rotation. Two patients (4.88%) met these criteria and subsequently required revision surgery. There were no cases of arthrofibrosis or secondary ACL/PCL failure during the follow-up period. One patient (2.44%) developed superficial infection secondary to fat necrosis, which resolved following surgical debridement and intravenous antibiotics without the need for further intervention. Sensory deficit in the form of numbness over the anteromedial aspect of the leg was reported by five patients (12.19%), consistent with transient irritation of the saphenous nerve branch, which recovered over time in all cases.

## Discussion

The findings of this study demonstrate that acute anatomical repair of femoral-sided MCL and PMC injuries in the context of MLKI using the double shoelace construct results in good to excellent long-term functional outcomes. Patients achieved significant and sustained improvement in Lysholm and IKDC scores over a minimum follow-up duration of 10 years, accompanied by a high rate of return to preinjury levels of sports participation and a low incidence of surgical failure or postoperative arthrofibrosis.

Historically, the management of complex MCL injuries has been influenced by concerns regarding postoperative stiffness and arthrofibrosis resulting from prolonged immobilisation in casts or braces and non-anatomical repair techniques [9,10]. These concerns have led many surgeons to advocate delayed treatment strategies, often without considering the anatomical site or severity of the tear. However, femoral- or tibial-side avulsion tears of the MCL demonstrate inferior intrinsic healing capacity and are increasingly recognised as injuries that benefit from early surgical repair, particularly when associated with ACL or PCL reconstruction in MLKI. Moreover, the POL contributes substantially to posteromedial stability and has been reported to be injured in up to 99% of patients with AMRI [11]. Contemporary literature emphasises that significant involvement of the PMC and particularly the POL may result in persistent AMRI despite repair of the superficial MCL alone, thereby necessitating a more aggressive and anatomically comprehensive approach [12,13].

Failure to address PMC injuries adequately can result in persistent valgus and rotational laxity, delayed return to sport, progressive intra-articular damage, and inferior long-term outcomes [4,14]. While reconstruction remains an option, it carries inherent disadvantages, including donor site morbidity, increased risk of postoperative infection, tunnel collision with concomitant ligament reconstructions, and loss of bone stock [15]. In contrast, acute anatomical repair, where soft tissue planes remain definable, offers the advantage of restoring native knee biomechanics while minimising surgical morbidity.

Several clinical series have evaluated acute repair of MCL and PMC injuries and reported postoperative Lysholm scores in the 90-98 range with low rates of arthrofibrosis and failure, which is similar to the functional results in the current cohort [16-22]. Distal grade III MCL avulsion repairs in multiligament settings have yielded mean Lysholm scores above 90 with high activity levels on the Tegner scale, while combined MCL-PMC repairs have restored coronal plane stability with IKDC scores in the low to mid-80s [16-25]. These data support the concept that anatomically directed repair of identifiable medial lesions can achieve outcomes comparable to reconstruction in appropriately selected patients (Table 3). 

**Table 3:** Comparative clinical outcomes of medial collateral ligament repair in MLKIs in the literature P: proximal; M: midsubstance; D: distal; MLKI: multiligament knee injury; ACL: anterior cruciate ligament; ND: not documented

Study (Author(s), year)	Number of Patients with MCL injury	Age (years)	Site of MCL injury			Injury to surgery time (weeks)	Mean Postoperative Lysholm Score at final follow-up	Mean Postoperative IKDC Score at final follow-up	Mean Postoperative Tegner Score at final follow-up	Total Number of			Period of follow-up (years)
			P	M	D					Arthrofibrosis	Surgical Failure	ACL Failures	
Shirakura et al. (2000) [17]	14	mean±SD, 31 ±13.7	11	0	2	<2	98.5	ND	5.2	0	0	0	mean±SD, 5.63 ± 2.5
Tzurbakis et al. (2006) [18]	17	mean±SD, 28.6±11.9	2	1	8	<2	90.3	ND	ND	2	1	0	range, 2-8
Osti et al. (2010) [19]	22	range, 18-39	0	22	0	>6	96	ND	ND	0	0	0	2
Pandey et al. (2017) [20]	20	mean (range), 36 (18-55)	1	8	11	<3	94.6	83.3	ND	6	0	0	mean (range), 2 (2-7.5)
Desai et al. (2020) [21]	20	mean (range), 22.7 (16-38)	0	0	20	5	91.5	88.8	7	2	0	0	2
Holuba et al. (2023) [22]	20	mean (range), 41.4 (28.5-48)	15	0	3	<6	95	82.2	5	1	2	4	2
Present study	41	mean (range), 35.12 (18-59)	41	0	0	<3	92.8	37.2	4.54	0	2	0	mean (range), 10 (10.35-13.9)

The double shoelace configuration described in this study is designed to capture a broad soft-tissue footprint of the sMCL and POL at their femoral attachment, thereby distributing tensile forces over a wider area and reducing the risk of suture cut-through. By sequentially advancing the anterior margin of the PMC to the sMCL in opposing shoelace passes, the construct restores both valgus restraint and anteromedial rotatory stability while respecting the anatomic isometric point on the medial femoral condyle. This robust fixation permits early controlled mobilization in a hinged brace, potentially lowering the incidence of arthrofibrosis that historically tempered enthusiasm for aggressive medial repair in MLKIs [2,15,25].

In the present cohort, almost all preinjury athletes were able to resume competitive sports at approximately 10 months after surgery, which compares favourably with published return-to-sport rates after multiligament knee reconstruction in young, active patients. Early single-stage treatment of cruciate and medial-sided injuries using an anatomic repair strategy may shorten overall rehabilitation, limit secondary intra-articular damage, and reduce the risk of cruciate graft failure that can occur when residual valgus and anteromedial rotatory laxity are left untreated. Consequently, a low threshold for operative management of clearly defined femoral-side MCL and PMC injuries appears justified in high-demand individuals who require reliable restoration of stability and a realistic prospect of returning to pivoting or contact sports [26-30].​​

Despite these encouraging results with 10 years of follow-up, important uncertainties persist regarding the optimal indications and patient selection criteria for medial repair versus reconstruction in the context of MLKI. The decision-making process remains complex, influenced by injury chronicity, tissue quality, associated ligament involvement, and patient activity demands. Well-designed prospective comparative studies with larger sample sizes, standardized return-to-sport outcome measures, instrumented laxity assessments, and complementary biomechanical analyses are essential to clarify whether repair constructs such as the “double shoelace repair” techniques offer distinct advantages over other repair strategies or medial ligament reconstructions across varying MLKI patterns and functional expectations.

Our study is not without limitations. The retrospective design, with the absence of a control group or a comparative reconstruction cohort, limits the ability to establish definitive causal relationships. The small sample size (n=41) restricts generalizability to a more diverse population. Another limitation is the lack of objective laxity measurements or biomechanical validation to provide quantitative support for the clinical outcomes.

## Conclusions

The “double shoelace repair” technique for acute femoral-sided MCL and PMC injuries in the setting of MLKI provides dependable restoration of medial and rotatory stability, excellent long-term functional outcomes, and high rates of return to preinjury activity with a low complication profile. By adhering to anatomic repair principles, this method preserves native tissue biomechanics, enables early structured rehabilitation, and avoids several disadvantages associated with ligament reconstruction in suitably selected acute cases.

The results of this study reinforce the role of early operative intervention for proximal avulsion patterns accompanied by anteromedial rotatory instability, particularly among young and active individuals. At the same time, the findings highlight the need for well-designed prospective comparative studies to refine surgical indications, validate technique-specific advantages, and optimise treatment algorithms for MLKIs.
